# When Care Becomes Harm: Unmasking Covert Abuse in a Non-Verbal Adult with Autism

**DOI:** 10.1192/j.eurpsy.2026.11254

**Published:** 2026-07-31

**Authors:** F. Bayam, R. Alves, M. Menino, S. Meleiro, I. Marques, I. Franco, J. Melim, C. Almeida, C. Bayam, C. Laureano

**Affiliations:** 1 Serviço de Psiquiatria e Saúde Mental, Unidade Local de Saúde da Região de Leiria, Leiria; 2 Pedopsiquiatria, Unidade Local de Saúde de Coimbra, Coimbra, Portugal

## Abstract

**Introduction:**

Factitious Disorder Imposed on Another (FDIA), formerly Münchausen Syndrome by Proxy, is a covert form of abuse where a caregiver fabricates, exaggerates, or induces illness in a dependent to assume medical indispensability. Although mainly described in children, FDIA in adults with severe neurodevelopmental disorders is underrecognized. Adults with Level 3 Autism Spectrum Disorder (ASD) are especially vulnerable due to profound communication and autonomy deficits. Detection is further obscured when the perpetrator is a healthcare professional, whose authority may delay safeguarding. These cases raise significant diagnostic, ethical, and medico-legal challenges requiring multidisciplinary action.

**Objectives:**

To present a case of suspected FDIA in a non-verbal adult with profound ASD, and examine psychiatric, forensic, and safeguarding implications of medically mediated abuse.

**Methods:**

A 20-year-old male with Level 3 ASD and profound intellectual disability, fully dependent and non-verbal, was under the exclusive care of his mother, a registered nurse. He presented repeatedly with severe behavioral dysregulation, agitation, self-injury, aggression, consistently following abrupt, unsupervised medication changes initiated by the mother. Data from multiple admissions were reviewed, including behavioral assessments, treatment adherence, and staff observations during periods of guideline-based care free from caregiver interference.

**Results:**

Behavioral stabilization was consistently achieved during hospitalizations, under structured psychopharmacological regimen. Nursing staff documented repeated intrusive anorectal and genital manipulations by the mother, justified as relief of presumed fecal impaction, despite normal bowel function and absence of constipation. The mother also interfered with treatment administration, exaggerated symptoms, and undermined medical advice. Comprehensive evaluation, including labs, imaging, and psychiatric review found no medical cause for symptom fluctuations, strongly supporting exogenous induction or fabrication.

**Conclusions:**

This case demonstrates FDIA in a non-verbal adult with severe ASD, a population at extreme risk of caregiver abuse. The patient’s inability to self-report and the mother’s professional authority delayed recognition. Recurrent destabilizations linked to unsupervised treatment alterations and inappropriate intrusions met criteria for FDIA. FDIA in adults with neurodevelopmental disorders is likely underdetected. Clinician vigilance is crucial when caregiver narratives dominate without objective evidence. Early multidisciplinary involvement, psychiatry, forensic services, safeguarding teams, legal counsel, is essential for protection. Institutional awareness, staff training, and structured safeguarding protocols are critical to identify and manage medically mediated abuse in this highly vulnerable group.

**Disclosure of Interest:**

None Declared

